# Heparin-Induced Hyperkalemia Assessment Utilizing the Naranjo Adverse Drug Reaction Probability Scale: A 40-Year Systematic Review

**DOI:** 10.3390/pharmacy13020055

**Published:** 2025-04-11

**Authors:** Divita Singh, Omnia A. E. A. Mesalhy, Michael J. Cawley

**Affiliations:** 1 Department of Pharmacy Practice, Temple University School of Pharmacy, Philadelphia, PA 19125, USA; michael.cawley0001@temple.edu; 2 Ain-Shams University Hospitals, Cairo 11566, Egypt; doctor_omnia25@yahoo.com

**Keywords:** hyperkalemia, heparin, low-molecular-weight heparin, adverse drug reaction, unfractionated heparin, hypoaldosteronism

## Abstract

Background: Adverse drug reactions have been reported as leading causes of morbidity and mortality. Unfractionated heparin- and low-molecular-weight heparin-induced hyperkalemia are side effects that have been reported in approximately 7 to 8% of heparin-treated patients. Algorithms, assessment tools, and decision aids are needed to assist in determining the causality of these adverse drug reactions. Aim: The aim of this study was to determine the number of case reports of hyperkalemia resulting from unfractionated heparin or low-molecular-weight heparin use by utilizing the Naranjo Adverse Drug Reaction Probability Scale. Methods: PubMed, International Pharmaceutical Abstracts, and the Cochrane Library were searched for relevant publications. Search terms and Boolean operators, including “hyperkalemia AND heparin”, “hyperkalemia AND low molecular weight heparin”, “heparin AND hypoaldosteronism”, and “low molecular weight heparin AND hypoaldosteronism”, were used. Searches were limited to case reports and human specimens. Results: A total of 29 case reports were identified, incorporating 38 patient cases. Of the 38 patient cases, 5 [4 involving unfractionated heparin and 1 involving low-molecular-weight heparin] (13.2%) utilized the Naranjo Adverse Drug Reaction Probability Scale to identify the possibility of an adverse drug reaction occurring due to exposure to unfractionated or low-molecular-weight heparin as probable. Conclusions: The available evidence suggests that clinicians’ use of the Naranjo Adverse Drug Reaction Probability Scale to determine the potential of hyperkalemia occurring due to exposure to unfractionated heparin and low-molecular-weight heparin is limited. Clinicians should be encouraged to utilize an objective monitoring tool to help standardize assessment of causality for all adverse drug reactions.

## 1. Introduction

Heparin is the oldest anticoagulant used in clinical medicine. Heparin is a naturally occurring polysaccharide belonging to the glycosaminoglycan family. After its discovery by McLean in 1916 as a naturally occurring polysaccharide, the drug went on to be marketed as a pharmaceutical product in the United States in the form of unfractionated heparin (UFH) in 1939 [1,2]. Since that time, decades of research have investigated the chemical structure, mechanism of action, and potential side-effect profile of this new class of anticoagulants. Successful experimentation resulted in the discovery of low-molecular-weight heparins (LMWHs) in the 1980s, which included enoxaparin and dalteparin, followed by Factor Xa Inhibitors, including fondaparinux, which was FDA-approved in 2000 [3,4].

Despite the extensive history of their use, UFH and LMWH exposure has resulted in adverse effects, such as bleeding, heparin-induced thrombocytopenia, and osteoporosis. In addition, hyperkalemia has also been reported as a rare toxicity effect [5]. Hyperkalemia is a potentially life-threatening electrolyte imbalance [5]. UFH- and LMWH-induced hyperkalemia are side effects that have been reported in approximately 7 to 8% of heparin-treated patients [5,6]. Although there is no internationally agreed upon definition of hyperkalemia, the European Resuscitation Council defines hyperkalemia as a plasma potassium concentration > 5.5 mEq/L [6]. Despite several other medications that are known to predispose patients to hyperkalemia, including angiotensin-converting enzyme inhibitors and non-steroidal anti-inflammatory drugs, UFH and LMWH are frequently overlooked as potential causes of hyperkalemia [5,6].

Adverse drug reactions (ADRs) are an important aspect of clinical practice. Many algorithms, assessment tools, and decision aids used to determine the potential of an ADR have been published, including the Yale, Karch, and Begaud algorithms; the Liverpool Adverse Drug Reaction Causality Assessment Tool (LADRCAT); the French Causality Assessment Method (FCAM); the World Health Organization–Uppsala Monitoring Center (WHO-UMC) criteria; and the Naranjo Adverse Drug Reaction Probability Scale (NADRPS) [7,8,9,10,11]. Although none of the ADR tools listed has been universally accepted as the gold standard, both the NADRPS and the WHO-UMC criteria are the generally accepted and most widely used methods in clinical practice.

The NADRPS was developed in 1981 at the University of Toronto; the tool was determined to demonstrate both reliability and validity in determining the causality of ADRs. The NADRPS consists of 10 questions concerning the implicated medication and the reaction. Each answered question receives an individual score, with the total score ranging from −4 to +13. The reaction is then quantified into one of four categories of likelihood that the drug was associated with the event. The reaction is considered definite if the score is +9 or higher, probable if it is 5 to 8, possible if it is 1 to 4, or doubtful if it is 0 or less [11].

Compared to the WHO-UMC system, causality assessment by the NADRPS requires additional information, including prior similar reports, administration of a specific antagonist, reaction occurrence in response to a placebo, the relationship between dose and event severity, any history of prior exposure, and confirmation through laboratory testing or direct clinical observation. In contrast, the WHO-UMC system primarily considers the clinical–pharmacological aspects of the case history, placing less emphasis on prior knowledge and statistical probability [8,11,12,13].

## 2. Aim

The purpose of this systematic review was to determine the number of case reports of hyperkalemia resulting from UFH and LMWH, utilizing the NADRPS. Our focus encompassed the use of UFH and LMWH over the past 40 years since the publication of the NADRPS.

## 3. Materials and Methods

This review was reported following the Preferred Reporting Items for Systematic Reviews and Meta-Analyses (PRISMA) guidelines, provided in File S1 [14]. This review was not registered.

### 3.1. Ethical Approval

No ethical approval was necessary, as review articles at our institution do not require approval.

### 3.2. Search Strategy

The search was limited to papers written in English involving human subjects and available as full texts. Case reports were identified to include patients treated with UFH and LMWH (enoxaparin and dalteparin) experiencing hyperkalemia with a serum potassium level > 5.5 mEq/L. Case reports without abstracts, case reports identified on or before the year 1981, clinical trials, randomized control trials, prospective trials, and comparative studies on the use of UFH or LMWH were excluded. In lieu of case reports without abstracts and not identified before the year 1981, the other study methodologies were excluded for a variety of reasons, including their definitions of hyperkalemia, serum potassium data not being available, and hyperkalemia not being identified as a study endpoint. A search strategy utilized databases that would identify the potential of hyperkalemia induced by exposure to UFH or LMWH. Each author conducted an independent literature search utilizing three databases: PubMed (1939 to June 2023), International Pharmaceutical Abstracts (1970 to June 2023), and the Cochrane Library (1992 to June 2023). Search terms and Boolean operators, including “hyperkalemia AND heparin”, “hyperkalemia AND low molecular weight heparin”, “heparin AND hypoaldosteronism”, and “low molecular weight heparin AND hypoaldosteronism”, were used. MeSH terms were not preferred as a search method, since not all content in PubMed is from MEDLINE, so not all articles have MeSH terms. In addition, the authors believed that using Boolean operators (AND) made our search much more precise. The following data were extracted for each study: author, publication year, patient case demographics (age/sex), UFH and LMWH dosing, days/hours of UFH and LMWH exposure, highest reported serum potassium concentration, other drugs used during UFH and LMWH exposure, NADRPS use, and NADRPS recorded score. Any case reports identified on or before the year 1981 were excluded, since the NADRPS was published in 1981. Information relevant to the discussion but which did not meet the inclusion criteria is provided within the References section of the manuscript.

### 3.3. Study Selection

All authors compared the search strategies to verify the results obtained and screened all the abstracts that were downloaded for review together to ensure that they met the inclusion and exclusion criteria. Any differences in data that were identified were discussed by mutual agreement between all three reviewers.

## 4. Results

### 4.1. Literature Review

**Figure 1:**
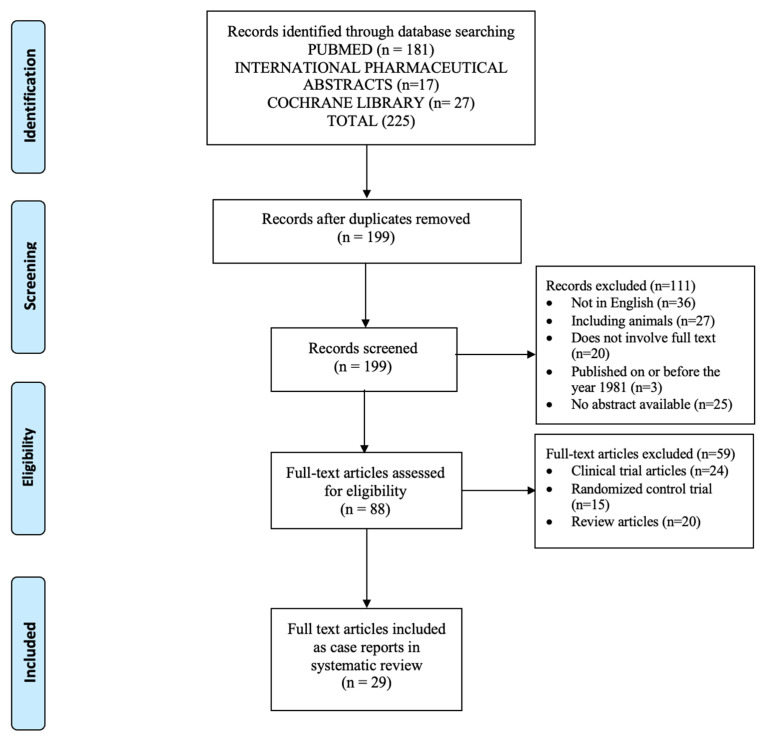
PRISMA flow diagram of studies selected for review [14].

The initial search provided 225 articles (Figure 1) [14]. After the removal of duplicate titles, 199 records were screened for review. After further screening, 170 records were excluded due to their meeting the exclusion criteria of the study. A total of 29 articles met the inclusion criteria. A summary of the 29 full-text articles, including a total of 38 case reports, is provided in Table 1 and Table 2. Of the 38 case reports reviewed, 31 case reports included UFH and 7 case reports included LMWH [15,16,17,18,19,20,21,22,23,24,25,26,27,28,29,30,31,32,33,34,35,36,37,38,39,40,41,42,43].

### 4.2. Unfractionated Heparin

Of the 31 case reports with UFH, 14 patients were geriatric (age > 65 years old), while 15 were adults younger than 65 years of age, 1 was a newborn infant of 28 weeks’ gestation, and 1 was a patient of unknown age, as the patient’s consent for publication was not obtained and therefore identifying information was not included [28]. Fourteen of the patients received heparin administration through the subcutaneous route, ten patients received it via the intravenous route, six received it via an unknown route, and one received it via both subcutaneous and intravenous routes of administration. The dose of heparin received varied, with 14 patients receiving 5000 units subcutaneously 2 to 3 times daily and 2 patients receiving 5000 units IV, then 1000 units per hour. Six of the case reports did not include a documented heparin dose [24,25,31,35,37,38]. The highest concentration of potassium reported varied from 5.8 to 9.2 mEq/L. Eighteen case reports included a list of other medications used concurrently which may have contributed to the patients’ hyperkalemia, while twelve case reports did not mention other medications being taken by the patients at the time. The NADRPS was utilized by only four case reports, all of which resulted in a score categorized as probable between 5 and 8 (probable ADR resulting from the identified medication (UFH)) [23,28,30,34]. Of the four case reports that utilized the NADRPS, two were for females, one was for a male, and one patient’s gender was unknown. The dosing and administration route varied among the four cases. In all cases utilizing the NADRPS, patients were on additional medications which could have contributed to their hyperkalemia. Of note, no other ADR assessment tool was noted to have been used in any of the cases.

### 4.3. Low-Molecular-Weight Heparin

Of the seven patients with LMWH use, five were geriatric [39,41,42,43]. Six patients received enoxaparin, while one patient received dalteparin. Five patients received an enoxaparin dose of 20 or 40 units SC daily, while one patient received an unknown enoxaparin dose and one patient received an unknown dalteparin dose. The duration of therapy with LMWH use ranged from 5 to 58 days. The highest concentration of potassium ranged from 6.1 to 7.1 mEq/L. Four patients received concurrent medications which may have contributed to their hyperkalemia, while three case reports did not specify the use of concurrent medications. The NADRPS was used in only one case report, resulting in a score of 6, categorized as probable [42]. Of note, no other ADR assessment tool was noted to have been used in any of the cases.

## 5. Discussion

### 5.1. Statement of Key Findings

Hyperkalemia may be attributed to non-pharmacological or pharmacological agents. Heparinoid products, including UFH and LMWH, are often overlooked as potential etiologies of hyperkalemia. However, it is important to understand the differences in the pharmacological action and mechanisms of hyperkalemia induction. LMWHs are prepared via a controlled enzymatic cleavage of UFH and a depolarization reaction and have been identified as having fewer side effects, a lower risk of bleeding, a longer half-life, a longer duration of action, and higher bioavailability [3,4]. Despite their beneficial side-effect profiles, UFH and LMWH have both been noted to induce hyperkalemia by impairing the excretion of potassium through suppression of aldosterone by inhibiting 18-hydroxylase enzymes (hypoaldosteronism). In addition, both agents may reduce the number and affinity of angiotensin II receptors in the adrenal zona glomerulosa and induce a progressive atrophy of the zona glomerulosa [5,6,44,45].

Hyperkalemia induced by UFH and LMWH occurs within 7 days of therapy and may occur at any dosage and via any route of administration, with potassium concentrations returning to normal within 1 to 3 days upon discontinuation of the instigating drug [4,5,6,44,45]. This process is especially noticeable in elderly patients, diabetic patients, and patients with kidney failure who are unable to adequately compensate (i.e., through increased renin production) to maintain normal potassium values, with 45% of patients receiving heparin and 71% of patients receiving LMWH in the population considered as geriatric [15,16,17,22,26,27,29,31,33,35,36,37,38,39,41,42,43,46]. Additionally, we noted hyperkalemia in patients who received varying doses of UFH by different routes of administration and varying doses of LMWH.

Through our review, we identified that the majority of cases reporting UFH- or LMWH-induced hyperkalemia failed to use the NADRPS, with only 13% of the cases with UFH and 14% of the cases with LMWH using one of the most widely utilized ADR causality assessment tools in clinical practice [23,27,30,34,43]. Based on our review, all of the cases which used the NADRPS resulted in a score between 5 and 8, categorized as a probable likelihood that the UFH or LMWH was associated with hyperkalemia [23,28,30,34,43]. Of note, in all cases where NADRPS was utilized, patients were on concurrent medications which could have contributed to hyperkalemia [23,28,30,34,43].

The authors chose to use the NADRPS over other assessment tools due to its being one of the most common tools used in North America. Also, data have shown that the NADPRS has similar scoring to other methods. A major advantage is its simplicity, as it includes only 10 questions compared to other methods that include 57 questions [47].

Although 5 of the patient cases identified the possibility of an ADR occurring due to exposure to UFH or LMWH, the remaining 33 cases did not identify any form of ADR algorithm, assessment tool, or decision aid to support the causality of an ADR. The authors of these cases may have hypothesized that hyperkalemia may have been associated with exposure and timing of UFH or LMWH dosing, patient clinical findings, serial laboratory data, ruling out other pharmacological and non-pharmacological etiologies, and clinical judgment.

### 5.2. Weaknesses

This systematic review did have multiple limitations, including the NADRPS. One question within the NADRPS is tailored and designed to be used within a controlled trial: “Did the adverse reaction reappear upon administration of placebo?”. Many clinicians would answer “no”, thus lowering the score of the ADR event. The result may drop the score by one, thus swaying the results from a probable to a possible score. Even though the NADRPS has demonstrated reliability and validity in its original publication, there are inherent flaws in interpretations of using the tool. If a potential ADR may have occurred due to a potential drug–drug interaction rather than due to a single drug, the NADPRS would need to be applied to each of the potential drug causes, which was not performed by the authors in our review. One of the limitations is that the tool was not utilized on other potential culprit medications mentioned in the cases reported. So, it is impossible for the authors of this manuscript to determine if the NADRPS was applied for each potential drug cause. Also, due to variant elements of publication bias in utilizing the NADRPS, there is always the possibility of individual interpretation of the results. Naranjo et al. utilized strict definitions of a potential ADR, considering both physicians (two) and pharmacists (four) who used it as a tool, and reviewed 63 randomly alleged ADRs in five leading medical journals [10]. The authors of the case reports included in this review may have utilized different definitions of ADR, and subjective biases of the many different clinicians may have determined whether an ADR was deemed to have occurred.

Risk assessment bias is traditionally used to evaluate case reports, since they are considered uncontrolled studies and, therefore, due to methodological design, may have inherent flaws of greater risk of bias. Based upon this assumption, qualitative tools are needed to assess their methodological quality. The Critical Appraisal Skills Programme (CASP) tool is one of the tools most used to assess health-related quality evidence [48]. The CASP utilizes a checklist strategy to critically appraise case reports to assess the validity of the results and their usefulness in decision making [49]. Although this tool may have been utilized to better assess the quality of our case-report evidence, the authors did not utilize the tool, since there is limited existing guidance on its application.

The case reports were also inconsistent in discussing other non-drug causes of hyperkalemia, including bleeding, poor renal function, trauma, rhabdomyolysis, acidosis, and dehydration. Having a better understanding of non-drug-induced causes of hyperkalemia would have provided better clarity regarding whether a case of hyperkalemia had a pathological or physiological cause.

Language bias is another limitation of the study, as only English-language articles were included, which limited our results. Also, no randomized clinical trials, prospective studies, or comparative studies were included.

### 5.3. Further Research

The majority of cases reporting UFH- or LMWH-induced hyperkalemia precluded the NADRPS, one of the most widely utilized ADR causality assessment tools in clinical practice. With the prevalent use of heparin products, it is imperative that we understand the probability of ADRs associated with their use. Moving forward, it is crucial that we incorporate the use of accepted assessment tools to objectively identify the risk of certain ADRs attributed to a certain medication. Having comprehensive knowledge of the ADR risk associated with a medication could help us proactively monitor for such side effects in clinical practice.

## 6. Conclusions

This review identified the current literature of case reports of hyperkalemia associated with the use of UFH and LMWH utilizing the NADRPS. Available evidence suggests that clinicians’ use of validated assessment tools such as the NADRPS to determine the potential of hyperkalemia occurring due to exposure to UFH and LMWH is very limited. Clinicians should receive training and be encouraged to utilize an objective monitoring tool such as the NADRPS to help standardize assessment of causality for all adverse drug reactions and report them to national pharmacovigilance centers.

## Figures and Tables

**Table 1:** Summary of published reports of hyperkalemia induced by exposure to unfractionated heparin.

Author	Year	Age/Sex	Full Patient History	UFH Dosing	Duration of Therapy (Days)	Highest Concentration of Potassium (mEq/L)	Time in Days Until K Normalized (<5.5)	Relevant Concurrent Medications and Substances Used During UFH Dosing	Naranjo Scale (Yes/No) Score	Calculated Naranjo Total Score	Naranjo Score Interpretation
Brohee D [15]	1984	92 years old/female	Unknown	5000 U IV q 4 hr	4	6.4	Unknown	Unknown	No	NA	NA
				12,500 U SC BID	6					NA	NA
				5000 U SC TID	Unknown					NA	NA
Edes TE [16]	1985	58 years old/male	Paraparesis	5000 U SC TID	27	7.5	3	Unknown	No	NA	NA
		51 years old/male	DM	5000 U SC TID	7	6.8	5	Unknown	No	NA	NA
		87 years old/male	CAD	5000 U SC TID	7	6.5	5	Unknown	No	NA	NA
		63 years old/female	DM2, HTN, and CHF	5000 U SC BID	10	6.3	5	Furosemide 40 mg IV every morning, changed to PO after 3 days. Potassium chloride 20 mEq BID for 3 days, then potassium containing salt for 3 days, and then discontinued	No	NA	NA
Busch EH [17]	1987	77 years old/male	DM and HTN	5000 U SC BID	14	8.3	Unknown	Unknown	No	NA	NA
		35 years old/male	DM and HTN	7500 U IV followed by 1000 U/hr	10	7.6	Unknown		No	NA	NA
		57 years old/female	DM	5000 U SC BID	5	6.1	Unknown	IV antibiotics	No	NA	NA
Aull L [18]	1990	49 years old/male	Morbid obesity; jejunoileal bypass, followed by conversion to a vertical banded gastric partitioning and a gastric bypass; and, eventually, enterolysis and herniorrhaphy	5000 U SC BID	52	6.7	1	Cefotetan 1 g IV for two doses, ranitidine 50 mg IV q8 h, patient-controlled morphine, amphotericin B 325 mg, mycostatin 500,000 U swish and swallow q8 h, albuterol inhaler 2 puffs q4 h, ranitidine 200 mg/day, and potassium 20 mEq/day	No	NA	NA
		41 years old/female	DM; hysterectomy; and right-sided, below-knee amputation	5000 U SC BID	51	6.2	1	Patient-controlled morphine, sliding-scale insulin, gentamicin, clindamycin, and ampicillin	No	NA	NA
Mitchell S [19]	1993	65 years old/male	COPD, CABG, CHF, stroke, BPH, renal calculus, and a non-functioning left kidney	5000 U IV, followed by 1400 U/hr, stopped on day 24, then resumed on day 27 with increased dose	50	5.8	Unknown	Acetylcysteine 650 mg po daily; nitroglycerin 0.3 mg SL every evening; salbutamol and ipratropium metered-dose inhalers 2 puffs QID; diltiazem 90 mg po BID; cotrimoxazole po BID; prednisone 15 mg po daily, decreased by 5 mg every 2 days and discontinued; and magnesium hydroxide, docusate sodium, and aluminum hydroxide and magnesium hydroxide suspension prn	No	NA	NA
				Unknown	2	5.3	NA	Unknown	No	NA	NA
Bacon NC [20]	1997	56 years old/male	HTN and CKD	5000 U IV, then 1000 U/hr	10	6.4	Unknown	Erythropoietin, alfacalcidol, calcium carbonate, isosorbide mononitrate, aspirin, and atenolol	No	NA	NA
Preston RA [21]	1998	42 years old/male	AIDS	5000 U IV, then 1000 U/hr	5	8.1	1	Sulfamethoxazole-trimethoprim 5 mg/kg every 8 h	No	NA	NA
Orlando MP [22]	2000	68 years old/female	HTN, tobacco use, and alcohol abuse	5000 U SC BID	6	5.3	NA	Sucralfate 1 g, ticlopidine hydrochloride 250 mg, and nifedipine 30 mg daily	No	NA	NA
				5000 U SC TID	7	6.4	2	Sulfamethoxazole-trimethoprim for 7 days at a renally adjusted dose	No	NA	NA
				5000 U SC TID	8	6.4	2	Sulfamethoxazole-trimethoprim BID	No	NA	NA
				5000 U SC BID	5	5.4	NA	Unknown	No	NA	NA
		70 years old/male	DM and curative rectal cancer surgery	5000 U SC BID	18	6.5	3	Sulfamethoxazole-trimethoprim BID, sodium polystyrene sulfonate, glucose, and IV fluids	No	NA	NA
				5000 U SC BID	4	5.7	2	Unknown	No	NA	NA
Sherman DS [23]	2000	34 years old/male	No significant history	7000 U IV, followed by 1600 U/hr; decreased to 1200 U/hr	37	6.4	1	Total parenteral nutrition containing potassium 42 mEq/d, insulin drip, and intermittent fludrocortisone use	Yes	Not provided	Probable
Day JR [24]	2002	55 years old/female	MI, HTN, and hypercholesterolemia	Unknown	1.5	7.1	2	Unknown	No	NA	NA
Su HM [25]	2005	40 years old/male	ESRD, DM, and HTN	Unknown	10	9.2	Unknown	Unknown	No	NA	NA
Thomas CM [26]	2008	85 years old/female	DM2 and peripheral neuropathy	5000 U SC BID	4	6.1	1	Benzylpencillin 1.2 g q6h for 2 days and then amoxycillin 500 mg PO TID for 8 days	No	NA	NA
Liu AA [27]	2009	75 years old/female	CHF, dyslipidemia, and DM2	5000 U SC BID	8	6.9	2	Aspirin 100 mg, atorvastatin 20 mg, carvedilol 12.5 mg BID, and insulin aspart protamine and insulin aspart (30/70) 26 units and 8 units	No	NA	NA
Brown G [28]	2011	Unknown	Unknown	10,000 SC TID	15	6.2	1	Potassium included in enteral nutrition solution; sodium polystyrene sulfonate 30 g rectally on day 13; then, PO on days 14 and 15 and fludrocortisone 0.1 mg PO daily on days 15, 16, and 17	Yes	6	Probable
Bhaskar B [29]	2012	68 years old/male	DM2, ARD, and hypercholesterolemia	200 U/kg IV, followed by a second dose of 50 U/kg	1 hr	6.8	1	Unknown	No	NA	NA
Cho R [30]	2013	52 years old/male	No significant history	4700 U IV, followed by 18 U/kg/hr	3 hrs	5.8	2	Furosemide 20 mg, metoprolol tartrate 10 mg IV q4 h, hydrocortisone 100 mg IV q8 h, and methimazole 20 mg PO q6 h	Yes	Not provided	Probable
Sodhi K [31]	2013	26 years old/male	Asthma and addiction (tramadol, diclofenac, heparin pheniramine, and dexamethasone)	Unknown	Unknown	9.1	NA—deceased	Ceftriaxone, followed by meropenem, teicoplanin, and ampicillin	No	NA	NA
Shimokaze T [32]	2014	Newborn infant, 28-week gestation/female	No significant history	55 IV U/kg/day	12	7.9	1	Breast milk, indomethacin IV, and Lactobacillus casei PO	No	NA	NA
Zhou B [33]	2016	68 years old/female	HTN, nephrolithotomy in both sides, and DM	43,750 U IV	3	6.66	1	Telmisartan 80 mg daily, lacidipine 4 mg daily, gliclazide 30 mg, and metformin 500 mg BID	No	NA	NA
Custodio M [34]	2021	47 years old/male	HTN, stroke, and DM	5000 U SC TID	8	6	2	Piperacillin–tazobactam and linezolid, insulin, albuterol, and furosemide	Yes	6	Probable
Baleguli V [35]	2021	72 years old/male	CKD and leukemia	Unknown	6	6.7	1	Unknown	No	NA	NA
Nlandu YM [36]	2022	72 years old/male	DM2, HTN, and unspecific arrhythmia	Unknown	7	6.6	NA—deceased	Insulin, dextrose, β2 agonist, and sodium polystyrene sulfonate	No	NA	NA
Kovacs J [37]	2022	77 years old/male	MI, DM2, BPH, CKD, and nephrectomy	Unknown	4	6.2	1	Unknown	No	NA	NA
				Unknown	2	6	1		No	NA	NA
Kargar F [38]	2023	69 years old/female	CAD, ischemic heart disease, HTN, DM, dyslipidemia, and AF	Unknown	6 hrs	5.8	NA—deceased	Unknown	No	NA	NA

Abbreviations: CAD, coronary artery disease; DM, diabetes mellitus; DM2, diabetes mellitus type II; HTN, hypertension; CHF, congestive heart failure; COPD, chronic obstructive pulmonary disease; CABG, coronary artery bypass graft; MI, myocardial infarction; AF, atrial fibrillation; BPH, benign prostatic hyperplasia; CKD, chronic kidney disease; ESRD: end-stage renal disease; ARD: acute renal disease; Unknown: not mentioned in article; NA: not applicable; UFH, unfractionated heparin; mEq, milliequivalent; L, liter; U, units; IV, intravenous; SC, subcutaneous; BID, twice a day; TID, three times a day; QID, four times a day; hr(s), hour(s); g, gram; mg, milligram; kg, kilogram; PO, by mouth; prn, as needed; SL, sublingual.

**Table 2:** Summary of published reports of hyperkalemia induced by exposure to low-molecular-weight heparin (LMWH).

Author	Year	Age/Sex	Full Patient History	LMWH Dosing	Duration of Therapy (Days)	Highest Concentration of Potassium (mEq/L)	Time in Days Until K Normalized (<5.5)	Relevant Concurrent Medications and Substances Used During LMWH Dosing	Naranjo Scale Mentioned (Yes/No)	Calculated Naranjo Total Score	Naranjo Score Interpretation
Wiggam MI [39]	1997	86 years old/female	Chronic pyelonephritis	Enoxaparin 20 mg SC daily	58	6.7	Unknown	Inhaled salbutamol 400 mcg daily	No	NA	NA
Rippin JD [40]	2003	45 years old/female	DM1, HTN, previous recurrent DVT, and seronegative RA	Dalteparin (dose unknown)	5	7.1	7	Warfarin, human insulin, colchicine, lansoprazole, and lisinopril	No	NA	NA
Danguy C [41]	2012	71 years old/male	DM	Enoxaparin 40 mg SC daily	6	6.1	4	Unknown	No	NA	NA
				Enoxaparin 20 mg SC daily	3	5.2	NA			NA	NA
		63 years old/female	DM	Enoxaparin 20 mg SC daily, increased to 40 mg after 4 days	14	6.1	2	Unknown	No	NA	NA
				Enoxaparin 40 mg SC daily	4	5.3	NA			NA	NA
		71 years old/female	Arterial HTN	Enoxaparin 40 mg SC daily	17	6.2	2	Unknown	No	NA	NA
				Enoxaparin 20 mg SC daily	3	5.4	NA			NA	NA
Scalese MJ [42]	2016	74 years old/male	Intellectual disability; right-sided, above-the-knee amputation; burn injury covering 27% of his body surface area; ileostomy; and PEG tube	Enoxaparin 40 mg SC daily	8	7	2	Ertapenem 1 g daily; metoprolol succinate 200 mg daily; lactulose 20 g daily; pantoprazole 40 mg daily; nutritional supplements; meal replacements—1 can three times daily (28.5 mEq potassium per day); and calorically dense, fiber-fortified therapeutic nutrition—1.2 L can three times daily	Yes	6	Probable
Landolfo M [43]	2023	80+ years old/male	Surgically treated bladder and prostate cancer	Enoxaparin (dose unknown)	10	7.1	2	IV corticosteroids, antibiotics, proton-pump inhibitor, and furosemide	No	NA	NA
										NA	NA

Abbreviations: DM, diabetes mellitus; DM1, diabetes mellitus type 1; HTN, hypertension; RA, rheumatic arthritis; DVT, deep vein thrombosis; PEG, percutaneous endoscopic gastrostomy; NA, not applicable; g, gram; mg, milligram; mcg, microgram; mEq, milliequivalent; L, liter; IV, intravenous; SC, subcutaneous; Unknown: not mentioned in article.

## Data Availability

The datasets generated during and/or analyzed during the current study are available from the corresponding author on reasonable request.

## Supplementary Materials

The following supporting information can be downloaded at https://www.mdpi.com/article/10.3390/pharmacy13020055/s1: File S1: PRISMA 2020 for Abstracts Checklist and PRISMA 2020 Checklist title [50].
